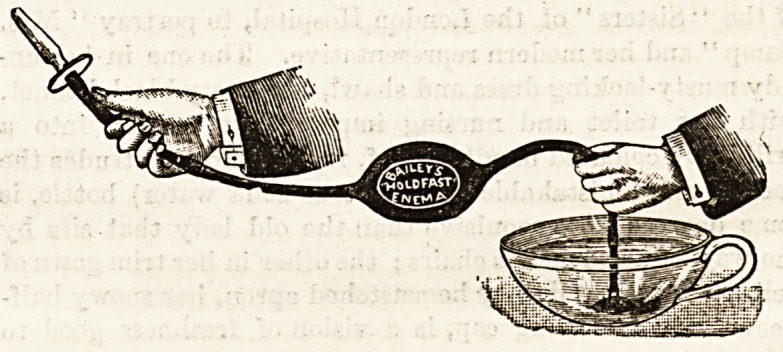# "The Hospital" Nursing Mirror

**Published:** 1897-05-29

**Authors:** 


### "The Hospital" Nursing Mirror.

Being the Nursing Section of "The Hospital."

[Contributions for this Section of "The Hospital" should be addressed to the Editor, The Hospital, 28 & 29, Southampton Street, Strand, London, W.C., and should have the word "Nursing" plainly written in left-hand top corner of the envelope.]

### News from the Nursing World.

### ROYALTY AT POPLAR.

The opening of the Blackwall Tunnel was a great occasion for the East-end of London; it had quite a little Jubilee all to itself, and the demonstrations of loyalty which greeted the Prince and Princess of Wales on Saturday last as they drove through the crowded streets of Whitechapel, Limehouse, and Poplar were very real and hearty. The Poplar Hospital for Accidents commanded an excellent view of the proceedings at the northern approach to the tunnel, and a big stand was erected outside the building and the seats sold for its benefit. The ward balconies were filled with eager patients, whose scarlet flannel jackets, relieved by the nurses' white aprons and caps, made an effective show of colour. The Royal party arrived at the entrance at half-past three, and in a few moments had disappeared into the tunnel; then the visitors to the hospital took the opportunity of going over the newly-furnished isolation block, and the delightful theatre and receiving-room just added to the building, and soon to be put into use.

### JUBILEE DAY AT ST. THOMAS'S.

Nurses at St. Thomas's Hospital will have an excellent view of the Jubilee procession, special provision having been made for some eighty of them on the steps of the Treasurer's house, which will ensure them a most commanding position. Old St. Thomas's nurses, with Miss Gordon's permission, are to be allowed to use the top outside corridor to view the procession so far as is possible from that point. The stands erected on the Westminster Bridge front of the hospital are to accommodate 1,400 people, and these, with the exception of a few complimentary tickets, have been all sold at five and three guineas apiece, so that the funds of the hospital will derive a good profit.

### A COMMEMORATION COT.

The people of Lambeth have already raised a sum of nearly £600 in aid of the Diamond Jubilee Cot which is to be endowed at the Royal Hospital for Women and Children, Waterloo Bridge Road, in commemoration of the Queen's reign. The sum total required to complete the endowment is £1,000.

### NORTH LONDON NURSING ASSOCIATION.

A sale of work on behalf of the funds of the North London Nursing Association, at the institution, was opened on the 19th inst. by Mrs. B. L. Cohen. Mr. Goulding, chairman of the Executive Committee, explained to the visitors something of the history of the association, and said that the present sale was being held to clear off a debt of £200. The stalls were filled with useful and pretty things, and there was a good attendance; visitors, it is to be hoped, coming, as Mrs. Cohen warned them they should come, with doubly-full purses. It is interesting to note, in the report of the nurses' work among the sick poor for the past year, that though no fees are asked, any thank-offerings are gladly received, and no less a sum than £16 5s. 10d. has been presented in small sums during this period, a sufficient proof of the estimate in which the association is held by those who benefit by its existence.

### THE SCIENTIFIC PRESS AT THE VICTORIAN EXHIBITION.

The Scientific Press had made full arrangements to render their stall in the Nursing Section at the Victorian Era Exhibition an interesting, instructive, and attractive exhibition, illustrative of the history, progress, and present position of nursing. Unfortunately, owing to a *contretemps* which could not have been foreseen, their plans have fallen through. The Scientific Press had intended, as a matter which it thought would be of interest to visitors from many parts of the world, to show nursing badges and medals collected from many institutions throughout the United Kingdom. Unfortunately, however, the promoters of the section from whom the stall was hired, for reasons best known to themselves, raised a special objection to an exhibition of any such badges or medals in the section, with the exception of those of the Queen's Jubilee Nurses. The narrowing of the scope of the so-called nursing division to exhibits which are practically representative of two institutions only placed the Scientific Press in the difficult position of having to make fresh arrangements at the last minute, and, finding that there was a probability of its exhibits being subject to still further interference, the Scientific Press has found it necessary, though with much regret, to withdraw from the exhibition. We regret that the nursing division, which might have been so representative an exhibition, if managed on public lines, should have been narrowed down to an advertisement of two institutions. The exhibition is, as we have said, interesting and well arranged, as far as it goes, but not of the comprehensive character worthy of the occasion.

### HOSPITAL LIFE IN ANCIENT GREECE.

A lecture on "Hospital Life in Ancient Greece" was given by Miss Jane E. Harrison, LL.D., at Lady Mary Egerton's house in Cadogan Square last week in aid of the fund of the Trained Nurses' Club, Buckingham Street. Miss Harrison's account of the methods of cure practised in the temples of Cheiro and Æsculapius, as made clear by ancient inscriptions recently excavated, many of them giving detailed particulars of symptoms and their alleviation, was exceedingly interesting, as the eager attention of her audience testified. The similarity between the cures by "hypnotic suggestion" of the present day and the cures of those ancient times in which "dreams" played a large part, showed that human nature after all is much the same now as in the days of Aristophanes. The lecture was well attended, and the club, we hope, will benefit. It meets, as our readers know, the needs of many nurses, supplying at an almost nominal subscription a pleasant central meeting and resting place, where nurses can rest and see the medical and nursing journals, and enjoy the advantages of a good lending library. With a subscription of only 5s. annually the club cannot, of course, be self-supporting, and any donations or subscriptions will be very gladly received by the President, Miss Wilson, 37, Barkston Gardens, S.W.

### A "VICTORIA HOME" FOR SWANSEA HOSPITAL.

At a large meeting held at Swansea the other day, it was unanimously decided to adopt as the Swansea "Jubilee" scheme the erection of a nurses' home at the hospital, where additional accommodation for the staff has been much needed for some time. Plans have been prepared, and the new building is to provide rooms for twenty-four nurses. If sufficient money is subscribed, it is hoped also to carry out an improved system of heating throughout the hospital. A resolution was passed calling upon the clergy of the neighbourhood to make special collections in aid of the scheme.

### A DISTRICT NURSE FOR WHALLEY.

A district nurse is to be established at Whalley, near Blackburn, in commemoration of the Queen's reign. A meeting was held the other day to discuss the project, at which a resolution in its favour was enthusiastically passed. A letter was read from Mrs. Worsley-Taylor promising £20 towards the fund and a yearly subscription of £10. The writer cordially approved of the scheme, and added that she thought there should be no difficulty in raising sufficient funds for the nurse's salary, especially after the people had learnt to appreciate the value of her services. Mr. R. Thompson, C.C., who presided at the meeting, wisely estimated the probable cost per annum of a well-trained nurse at not less than £100. Several substantial subscriptions were promised upon the spot, and the scheme was generally received with most encouraging approval.

### A HOME FOR SOLDIERS' WIDOWS.

At Kingston there is an excellent but little known institution, the Royal Cambridge Asylum for Soldiers' Widows. It was founded in memory of the late Duke of Cambridge, and in all its affairs the Duchess of Teck takes much personal interest. Of late years there has been considerable improvement in the arrangements for the sick and aged inmates, and a trained nurse has been appointed to the post of matron. Just now steps are being taken to build a proper sick ward, which is much wanted, and an entertainment room, for the occasional concerts which give so much pleasure to the old ladies, is also a needful addition, as also is a piano. Towards all these desirable things help will be very gladly received; and those who wish to know more of the institution, the only one of its kind in the kingdom, should go and visit it for themselves. They would have a cordial welcome from Miss Parry, the matron.

### SUNDERLAND NURSING INSTITUTE.

The annual meeting of the Sunderland Nursing Institute was held last month. The report, read by the hon. secretary, Dr. Larcombe, stated that the number of nurses on the staff at the end of the year was 15, and that the demand for their services continued to be far in advance of the supply. There was a deficiency on the year's work of £28 7s. 7d., the actual money earned amounting to £781 2s. 1d., and the expenditure to £809 9s. 8d. In accordance with instructions given at the last annual meeting, a district nursing committee had been formed, and the work of nursing among the poor in their own homes was being actively carried on. The two district nurses were housed at the institute, and their work supervised by the matron. In response to a question, Dr. Larcombe said he thought the association would this year receive some help from the Jubilee Fund Committee.

### NURSING IN ROME.

A correspondent tells us that private nurses are often wanted in Rome during the winter months, there being many English residents and frequently no nurses available but the Italian nuns. She suggests that one or two English nurses might find it worth while to go to Rome for the season, boarding in some pension as the most economical way of living, and believes plenty of work would be found. A great deal of caution must be exercised, however, by any nurses attempting such a venture, and full enquiry made beforehand. Those who have friends in Rome, or who could obtain introductions to the doctors there, might try and gain some thoroughly reliable advice on the subject, but we must once more repeat the warning that no nurse who cannot afford a possible failure to find patients or an indefinite waiting time should make such an attempt.

### GUY'S NURSES AT ATHENS.

Two interesting letters from Nurse E. M. Fox appear in the *Guy's Hospital Gazette* for May 22nd, giving a graphic description of her surroundings at the military hospitals at Athens, where she and several other English nurses are working amongst the Greek wounded. At first Nurse Fox and Nurse Davidson were sent to the military hospital, which is exclusively under the protection of the Crown Princess, a huge place with many wards of forty and fifty beds each, but later they were transferred to "l'Ecole Militaire," "a magnificent building, quite palatial in style, totally unsuited for the needs of a hospital," with marble floors and painted ceilings, and where the "beds look like cots in the vastness." Until the arrival of the English nurses there had been no women in the place at all, orderlies doing whatever was done, which apparently did not include the making of beds or the washing of the patients. On the evening of their arrival Nurse Davidson and Nurse Fox made 120 beds, "as a beginning," and the following day succeeded in obtaining a very small portion of water and having a general wash of faces and hands, greatly to the amusement of their patients. A change indeed from the orderly wards of a great London Hospital!

### SHORT ITEMS.

Two nurses have been dismissed from the Richmond Asylum, a patient under their care having been discovered by the medical officer with various injuries and bruises, for which the nurses in question were unable to give a satisfactory explanation.—The badge which is to distinguish the nurses of "Princess Christian's Army Nursing Reserve," besides bearing this designation round the edge, is stamped with a crown-surmounted cross, round which are representations of the rose, shamrock, thistle, and acorn.—The annual meeting of the Welsh branch of the Queen's Jubilee Nurses was held this month at the Town Hall, Cardiff.

### Pharmacy and Dispensing for Nurses.

By C. J. S. Thompson.

### XI.—PILLS AND THEIR EXCIPIENTS.

Pills are the usual method of administering drugs in solid form, and are very largely prescribed by medical practitioners, especially when they wish to give medicinal agents which cannot readily be given in solution. They are made of various sizes, to weigh from one to five grains. The ingredients prescribed in pills vary greatly, and beside the pill masses of the Pharmacopœia, combinations of extracts, gum resins and other similar bodies, alkaloids and active principles are met with frequently.

The first step necessary in compounding pills is to work the ingredients ordered into a plastic mass of suitable consistence, an operation which is sometimes attended with difficulty. This having been accomplished satisfactorily, the second stage is purely a mechanical one, and simply consists in rolling and cutting the mass and forming it into pills by the aid of the pill machine. To make a pill mass it is first necessary to combine the ingredients, and the medium which is usually added for this purpose is called the excipient.

In the choice of this excipient, in the majority of cases, lies the solution of the difficulties met with in the art of making pills. Occasionally pills are ordered to be made simply of soft extracts, as in the following instance: ℞. Ext. colocynth. comp., gr. ii.; ext. hyoscyam, gr. i.; ext. taraxaci, gr. ii.; misce fiat. pil. i. In this case it is necessary to add some inert powder, such as powdered marshmallow root, in order to form the extracts into a suitable mass. So dry as well as liquid excipients are necessary in pill-making, their use depending entirely on the nature of the ingredients ordered.

The procedure usually adopted in preparing a pill mass is to place the dry ingredients in the mortar first, and powder them as finely as possible. This is especially essential when crystalline substances such as sulphate of iron are being dealt with. Then carefully add sufficient of the excipient to make a mass, which should be adhesive enough to form a firm pill and just sufficiently soft to roll. In massing the ingredients the pestle should be used with a lever-like motion, in order to induce thorough incorporation. It is necessary that the dispenser should have a knowledge of the composition of the ingredients which she is working, in order to know the best excipient to employ. Thus, if none of the substances used contain a gum or other adhesive body, it is well the excipient should do so. On the other hand, should the ingredients be of a gummy or resinous nature, a liquid excipient may be used to bind the particles of powder into a solid mass. Care must be exercised not to use too much excipient, and so make the mass soft. The right amount can soon be judged with practice. When a very small quantity of an alkaloid such as strychnine or other powerful poison is ordered, it should be placed in the mortar and triturated with a little sugar of milk, in order to make sure it will be well distributed throughout the mass. When essential oils are prescribed, they should be the last ingredient to be placed in the mortar, and thoroughly well triturated with the other ingredients before massing. When the prescriber orders a certain excipient to be used, the dispenser should always employ it, except when he finds it is absolutely necessary to use another. If the prescriber leaves the size of the pill to the discretion of the dispenser, as shown in the following prescription: ℞. Hydrarg. subchlor. gr. $\frac{1}{6}$, fiat. pil. i., it should be made as small as possible with the aid of sugar of milk, liquorice, or althæa powder, and each pill when finished should not weigh more than one or two grains. To make the process as clear as possible to the student, we will describe how the following prescription for pills, which is a very common one, should be made: ℞. Ext. aloes, aquos., gr. ii; podophyllin resin, gr. $\frac{1}{6}$; euonymin, gr. i.; pulv. zingib, gr. ss.; misce fiat. pil. i.; mitte xii. In this case all the ingredients in the prescription are of a dry nature. The extract of aloes should first be reduced to fine powder, then the other ingredients added, and the whole thoroughly mixed. Compound decoction of aloes forms an excellent excipient when any preparation containing aloes is included among the ingredients; so in this instance, four or five drops of decoction of aloes placed in the mortar and well worked with the pestle will form an excellent mass. The mass, when finished, should present a perfectly homogeneous appearance throughout. Every particle should be scraped from the mortar and pestle. If crumbly or gritty it will not roll, and should be replaced in the mortar and worked up again with a little more of the excipient. It should be worked for a few moments between the fingers after being taken from the mortar to see it is sufficiently plastic, then placed on the board of the pill machine, which has been previously dusted over with a small quantity of powdered French chalk or magnesia to prevent sticking. With the flat side of the cutter, roll it out into a pipe of the required length, then place on the grooves and quickly cut into the number of pills ordered. A smooth surface and finish may be imparted by giving them a few rapid turns on the board under the pill finisher. They should be allowed to stand a short time to dry, after which they may be finished off by shaking them up with a small quantity of French chalk, lycopodium or magnesia; or they may be silvered, varnished, or coated with gelatine, French chalk or sugar, as desired.

### Excipients.

The nature of the excipient used in pill making necessarily depends on the composition of the drugs it is desired to mass. For bulky powders, such as ipecacuanha, jalap, and rhubarb, &c., simple syrup or treacle form a good medium. For resinous drugs, equal parts of spirit and acacia mucilage may be used, or when aloes is included the compound decoction forms an excellent mass. Where moisture is necessary the mucilage of tragacanth or acacia will be found useful. For dry powders that require the addition of an adhesive body to bind them, the confection of roses, or glycerine of tragacanth answer best. The latter is a most useful all round excipient, and very generally employed. It is of the consistence of a stiff jelly, and is made as follows: Take of powdered gum tragacanth, 1 drachm; glycerine, 4 ounces; water, 1½ drachms; rub them together in a mortar, then heat over a water bath for ten minutes, and allow to cool and set.

Among other useful excipients are powdered soap, which is employed for massing powdered opium, and with liquorice powder for making creasote pills. Bread crumb (which should be a day old) for massing calomel, and balsam of Peru, &c. Honey, for dry powders, and manna for nitrate of silver, calomel, &c. Confection of roses, for woody powders, but it must not be used with sulphate of iron or tannic acid. Kaolin ointment, for permanganate of potassium and nitrate of silver. Powdered gum acacia, tragacanth, liquorice powder, or althæa may be added to soft extracts to render the mass a suitable consistence.

There are also certain drugs which present special difficulties to the dispenser and which need special excipients, as for instance, carbolic acid, creasote, and phosphorus.

Such drugs require experience and careful manipulation to form into a good pill mass, and a great deal also depends on the method employed.

### Post-Graduate Clinics for Nurses.

By a Trained Nurse.

XV.—REFLECTIONS ON REST AND ARM-CHAIRS.

Where the real art of the nurse comes in is in making advantages out of drawbacks and comforts out of disadvantages. In setting forth suggestions for her patient's comfort I intend to dwell very lightly on how the nurse must insist on a lounge, a screen, and a bedside table, &c., being added to sick-room paraphernalia. For all this has been said many times before.

I will, however, try to set down some few precepts which have not yet become a threadbare story in the history of nursing so far as this has been written. So, while I cheerfully leave to many of those others who find complete satisfaction in adopting as their theme minute details of the exact position of medicine bottle and pill-box, or the angle at which a bedside table should assist in the economies of the sick-room, I will pass on to other things. Before doing so, this would, perhaps, be a good opportunity for reminding the private nurse that, in the ethics of the furnishing of a sick-room, the important item of a really soft, comfortable arm-chair *for the nurse* should by no means be forgotten. And this, by the way, is an article of furniture without which no hospital ward should be regarded as complete. Visions of "staff-nurses lounging in the wards" and "looking luxuriously idle in an easy chair," might easily be conjured up by some who would regard an easy chair in a hospital ward as a terrible act of heresy, directed against the root and foundation of the hospital creed as now formulated. But I do not believe that a staff-nurse could be so easily betrayed as to desert her path of duty because a comfortable chair was placed in that path. And that nurse only who was otherwise unworthy of a hospital post would "lounge unseemly" in a ward full of sick people. Broaching this simple reform to several hospital matrons—a comfortable chair in which tired nurses might take a real, restful half-hour when a lull in ward activity gives opportunity for recuperation and rest—I have been surprised to find that several such matrons really believe that an easy chair in a ward would mark the complete demoralisation and downfall of a nursing staff—so superficial and skin-deep, appears to some persons to be the dignity and self-respect of the average woman! Other matrons again—who, having a real knowledge of health and rest, realise what a terrible strain it is to young, developing women to stand about and bend about during so many consecutive hours of the day, to whom sitting down on the average chair of the hospital ward, hard, straight-backed, and unrestful to the highest degree, gives little comfort—have declared to me that they would gladly and cheerfully purchase out of their own purses an easy chair for the use of the nurses in every ward of their hospitals. "But," they said, "we are afraid an accusation of lack of discipline and propriety would be brought against us were we to carry out so good an intention." And reflecting on the relation between propriety and comfortable chairs, such a train of thought brings out most strongly that the traditional superstition still lives that where women are gathered together there should be an aroma of the penitentiary and a stern chastening of that desire so strong in human nature to combine a modicum of comfort with hard work. And it seems a pity that such a belief should not die and receive a decent burial in what a stump orator in Hyde Park recently so happily described as this "so-called nineteenth century." Thinking of the consultant who sits with perfect dignity in a particularly comfortable chair while prescribing for his patients, makes one wonder why a tired nurse might not retain the respect of her patients, despite an occasional comfortable "sit-down." And does not the matron keep up all the traditionary honour of her position while she interviews nurse candidates, and metes out even-handed justice to the erring of the staff, from the soft padded seat of her comfortable office chair? Why, then, the nurses in the ward should not be allowed in legitimate leisure moments to rest the aching tension of weary limbs and sinews strained by long standing, on the comfortable cushions of a soft chair, is a problem which has never become clear and logical to my consciousness. The private nurse, however, may, unrebuked and unchidden, sit in a comfortable attitude in a chair adapted for human restfulness, and will be the better nurse for the relief and rest she is permitted to take. The doctrine of harsh penance and ascetic self-punishment may be of enormous value to the idle and luxurious, but to the hard-worked nurse such severities are superfluous. And from my individual point of view it always appears to me that the person who appreciates some share of the comforts of this daily life is the most cheerful, pleasant sort of companion to have. At any rate, I would pray to be preserved when ill from the uncanny, severe sort of individual who "preferred on principle a straight-backed, hard, wooden chair!" Parenthetically, I might here commend a chair of just that build and description to be kept in every private sick-room. And its use should be restricted with perfect impartiality to those visitors of the sick person whose tendency it is to stay too long and talk too much! This device of the uncomfortable chair is that which I have found in actual practice to afford the most valuable method of limiting the visits of noisy and talkative persons whose idea of a "nice cheery little chat" with a sick person extends sometimes to an hour and a half! To devote the really nice, comfortable easy chair, which I am convinced Providence surely intended for the nurse, to such a visitor, is to bring down on the patient's head a babel of conversation which may retard by many days a pleasant, speedy convalescence.

Perhaps in no one particular can a nurse better prove her capacity for her work than in nicely balancing the atmosphere of the sick-room, so as to proportion the degrees of repose and cheerfulness, and thus put the patient under the best conditions for recovery. Many nurses can amuse, brighten, and cheer their patients. But all have not the faculty of resting the sick. And just as in the physician's treatment, a certain degree of stimulation—either by drug or diet—is suitable at one stage of an acute illness, while anodyne treatment may be necessary at another stage, so the nurse must learn by intuition and experience just how much mental stimulation and "brightening up" a sick person can stand with advantage. Often, from very weakness and weariness of body and mind, the sick will long for noise and racket to whip up their flagging forces, just as the nurse when tired longs most for a strong cup of tea; or the business man, with energies well-nigh spent, feels the need of a brandy-and-soda to carry on his work for the remaining hours he must needs spend "in the City." It is quite safe to leave to the text-books all the needful information as to the changing of the vitiated, used up air of a sick chamber. For my part I would prefer here to deal with the atmosphere of the sick-room in so far as this depends on the nurse herself. And as so much of this atmosphere hinges on the nature and temperament of the nurse, on her condition of health, and on her innate knowledge of the amount of rest, comfort, and healing she can diffuse into the air, it seems a matter worthy of consideration. Next week I shall deal further with this subject and quote some of the views Dr. Weir Mitchell expressed to me on one occasion as to a nurse who "rests her patient." In dealing later with the nursing of "rest cases" we shall see what an important bearing the attitude of the nurse has on the success of such cases.

### Nursing the Plague in India.

The following extracts from a letter of a nurse who is now at work at Poona will be interesting to our readers:—

"Sassoon Hospital, Poona, April, 1897.

"Cases (natives) for observation are sent here by troops from the station, but directly it is decided to be plague the cart is sent for and they are taken to the plague hospital. To tell you how they clasp my feet and beg me by all I hold dear to keep them here and not send them away is impossible; I can only say that my heart is frozen by their agony of terror. I am only allowed to keep European and Eurasian plague cases, and of course there is not accommodation for the others, but it is simply terrible. Some days I feel so tired and broken it is hard not to break down. . . . After death I must see to all the burning and disinfecting, for otherwise they would have no compunction about putting a new case on the same bed! . . .

"I went to nurse Dr. ——, who had a slight attack of plague, first going to his house and then bringing him here to the contagious wards. Now he is at Mahableshwar. A Mr. —— then came in (he is on some newspaper staff) with a huge bubo, and had to fight hard to get through. He was in a month except three days, and no one dared to hope he would live; head shaved and ice applications, and all along plenty of heart stimulation. With God's help he pulled through, a wreck, and now has gone to Kandala.

"In cases which almost without fail turn out to be plague the eyes are congested, there is terrible restlessness, and with a temperature of only 103 they are, as a rule, delirious.

"The fear is when the temperature falls, say, from 103 to 101·2, and two hours later your patient is dead. I had a case such as this. At a quarter to two he was as he had been all along, and his temperature had risen from 101·8 to 102. I went to him again at two o'clock, and saw the change; sent for the doctor, who injected ether and digitalis into the region of the heart, and also the next hour, but by half-past four he was dead. It is heart failure that causes death. A plague case requires a nurse all to itself for the first five days and nights after the temperature has dropped. Some doctors like the bubo painted with belladonna; others like it poulticed. As a rule, I think it has to be opened, and then, of course, poulticed; often it has to be touched up with blue stone. The patients are perfect wrecks when they get up and about again.

"In good cases the delirium yields to the ice bag, &c., but in unfavourable ones it increases in spite of the drop in temperature. The buboes appear in axilla, groin, neck, and abdomen; those in the abdomen are apparently the most agonising. The symptoms are terrible; agony in the abdomen, which it seems impossible to relieve; eyes starting out of head; very deep and rapid respiration and incessant gabbling; oppression on chest, &c. We apply mustard plasters (made with vinegar are the best) to abdomen, give morphia injections, and stimulate. These bad cases are usually fatal in twenty-four hours, death very sudden. Patients will walk about till within twenty minutes of their death if they are not watched. A characteristic of the disease, the same as with cholera, is mortal fright. They cannot help it.

"In every case there is a great deal of expectoration, which, but for the lack of the rusty colour, is like pneumonic expectoration. It almost seems to be one way of getting rid of the poison, and nurses must take great care to have some strong lotion in the receptacle, and in cases where the patient is unconscious the piece of lint used to wipe the sputum from the lips should be large and well soaked in 1 in 20 carbolic.

"As I have said, we are not allowed to nurse any but Europeans and Eurasians in the Sassoon Hospital, but the natives beg to be kept. They have to go to the Municipal Hospital."

### The Registration of Midwives.

The annual meeting of the Association for Promoting the Compulsory Registration of Midwives was held at the Vicarage Parish Room, Kensington, on Monday, May 28th, Lady Balfour, president of the association, in the chair. The Hon. Alan de Tatton Egerton, M.P., Dr. Foot, Miss Wilson, Mrs. Schwann, and others were on the platform, and there was a good attendance. Lady Jeune, who had promised to speak, was unfortunately unable to be present.

Mr. Egerton, who is in charge of the Midwives' Registration Bill, gave a cheerful report of its prospects, and was not without a faint trust that the second reading might even yet be reached before the end of the Session. He thought a suggestion made by Sir Blundell Maple might remove some of the opposition to the Bill, which arose from the feeling that it might deprive country practitioners of certain small fees which they looked upon as their own. Following on the lines of the Notification of Diseases Act, according to which every practitioner has to notify to the Sanitary Authority each case which comes under his care, and receives a certain fee from the local authority, Sir Blundell Maple suggested that every midwife should be required to notify each birth to the sanitary officer within 24 hours, the medical officer of health, or some qualified practitioner appointed by him, then visiting the case to see if it was being properly conducted, and receiving a minimum fee of 2s. 6d. If some such provision as this would really facilitate the passing of the Bill, it might be well to consider the advisability of its adoption.

Dr. Foot, who spoke from the point of view of a country practitioner, gave his own experience of the terrible mischief wrought in country districts by the ignorance of the women who called themselves midwives. He concluded by suggesting that every lady present should do her part towards promoting this necessary legislation by converting her neighbours, and, most important of all, her own medical man, to a right way of thinking on the subject.

Miss Wilson, in an excellent and stirring speech, put forward the urgent needs of the half million hapless mothers and infants whose lives were endangered by the present chaotic state of things, and compared the admirable working of the registration system in Switzerland, and Sweden and Norway, where she had herself studied it, with the utter disorganisation in this country. Surely Great Britain would not much longer lag behind other civilised European countries in protecting the lives of these poor women and their children. Dr. Cullingworth and Mrs. Schwann also addressed the meeting, which concluded with short statements from the Hon. Treasurer and Hon. Secretary.

### Royal British Nurses' Association.

### BREAY v. THE ASSOCIATION.

This case, wherein the plaintiff sought for an interim injunction to restrain the Association from applying its funds in defending the action of Fenwick v. De Pledge, came before Mr. Justice North on the 19th inst. The Judge gave no decision whatever on the merits of the case, but stated that there appeared to be a question as to the power of the Association to expend its funds as proposed, which would have to be determined at the trial. He therefore granted an interim injunction until the trial of the action, in order to prevent the plaintiff from being prejudiced. If the funds were expended there might be a difficulty in recovering the amount, even if the expenditure were held to be illegal. We understand that the defendants in this case are now taking it to the Court of Appeal.

### A Nurse's Visit to Texas.

IV.

Small-pox is a scourge in the southern cities. It will break out in Mexico, where the natives are such fatalists they will take no precautions whatever against it. In fact, when anyone falls ill with it all his friends go to see him, and some ladies who had spent the winter in the city of Mexico told me that women will even come to church with a child covered with the eruption in their arms.

Naturally, under these circumstances, it soon spreads across the border into Texas. Mexicans in San Antonio get it in their quarter, and as their American fellow townsmen are not so apathetic about it, and insist on the removal of the patients to the "pest camp" outside the town, they do their best to keep cases from the knowledge of the authorities and doctor them on some plan of their own, which is generally successful. The winter I was out it was raging in San Antonio, which was reported to look like a plague-stricken city, and from there it travelled north to Austin, brought by the railway cars the people said. Sometimes these are stopped running, that there may be no communication with the infected city.

When small-pox appears in a street a yellow flag is displayed, so that nervous folk may avoid passing that way. I tried to find out something about life in the "Pest Camp"—who nursed the patients, &c., but could not, being only assured that they had the best medical advice and care.

My brother was obliged to go to Austin to fetch stores for the Ranch during that epidemic, and as I was not nervous I went with him. I have often wished since that I had stayed in the town for a little while then, so as to have a better chance of gaining reliable information about that, and general nursing. When the warm weather came, and the people began to live more in the open air, the epidemic subsided, close packing and want of ventilation in the houses of the Mexicans, Indians, and Negroes, being in a large measure responsible for its severity.

Among the drawbacks to life in Texas, I must give the insects a very high place. They are a great trial to anyone whose skin is not abnormally thick, and their name is legion. There are tarantulas, centipedes, scorpions, poisonous spiders, stinging caterpillars, mosquitos, hornets, and ticks, the last being the most troublesome.

If you went for a walk you got them on you by scores off the grass and brush as you passed, and where they bit they stayed till pulled off, and it required a good pull too.

The hornets made their nests in the crevices of the roof woodwork, and sometimes when we sat in the verandah they would come out in such force that we were obliged to beat a retreat.

Besides all these there were plenty of daddy longlegs, which would run across your face as you were dropping off to sleep, mud-dobbers, which built little mud-nests in the corners of the rooms, and crickets and katydids in such numbers that after sunset the air was full of the sound of their chirping. But these were harmless plagues, that no one need object to.

In the summer time one had to keep a look out for rattle snakes. I did not see one all the seven months I was there, as I left early in the spring before they were much about. In cases of rattlesnake bite the settlers gave large quantities of brandy or whisky and applied ammonia to the wound, but they told me that unless the remedies could be applied at once the case was hopeless. Fortunately the warning rattle which the snake always gives as it coils itself to spring prevents this being a common accident.

I am afraid that so far I must have given the impression that I found Texas a place full of disagreeables, where all work and no play was the rule. It is quite time I mentioned some of the pleasures, of which I found a great many, but they must keep till my next article.

### Nursing in South Africa.

The *African Critic* is taking up the cause of nurses. We quote the following from a recent issue: "Another cry from hospital nurses in South Africa! In his report for 1896 on the New Somerset institution, the resident surgeon sets forth the grievances of the nursing staff—too much work and too little wages. They get no Saturday half-holidays and no Sunday off duty. Their annual leave is only twelve days during the first two years of service. They may have extra leave if ill-health demands it, but without pay. The salary is £30 per annum, with board, lodging, and uniform. The food allowance is none too liberal, and the sleeping space is only one small room for each couple of nurses. Often a nurse has to assist her widowed mother and younger brothers and sisters, and she cannot afford to take leave without pay. The doctor asks for discretionary powers in such cases, and also for authority to improve the diet scale in the matter of stimulants. Although the latter is a matter which requires delicate handling, the former should be at once dealt with on the lines advised. As one reads these details of Colonial philanthropic sweating, he can almost imagine the educated and trained nurse of to-day sighing for the freedom and high living of the days of rare old Sairey Gamp and Betsy Prigg. I said months ago, and now repeat, that English nurses should make careful enquiries before going to the Colonies."

### Appointments.

MATRONS.

Derbyshire Royal Infirmary. — Miss Elizabeth A. Wilkinson has been appointed Matron of this hospital. Miss Wilkinson was trained at the Leeds General Infirmary, and was subsequently promoted to the position of ward sister at that institution. She has more recently held the post of matron at the Women and Children's Hospital, Leeds, and that of lady superintendent of nurses at the North Staffordshire Infirmary, Stoke.

District Nurses' Home, 39, Great James Street, Londonderry.—Miss Susan Murphy has been appointed Superintendent of the Londonderry District Nurses' Home. Miss Murphy is a Queen's Nurse. She was trained at Crumpsall Infirmary, Manchester, and St. Bartholomew's Hospital, London, and received her district training at St. Patrick's Home, Dublin, where she was staff nurse for a year and a half.

Hammerwich Cottage Hospital.—Miss Annie Ker Frier has been appointed Matron to this hospital. Miss Frier was trained at the Edinburgh Royal Infirmary, and has since worked for more than two years on the staff of the Cambridge Private Nursing Institution.

### Wants and Workers.

"A Subscriber" sends us a piteous little appeal for indoor and outdoor toys and games for the Children's Convalescent Home, Plumpton, Sussex. The home is for poor Brighton children who need country air after illness, but it is little known and gets but a small meed of help. Mrs. Cracknell, the Matron, our correspondent says, will be most grateful for the smallest gift.

Nurse Helen, District Nurse, Cromhall, Falfield R.S.O., Gloucestershire, writes to ask if any reader can supply her with a book-rest to enable a paralysed patient to read in bed with comfort.

The Hon. Secretary of The Hospital Convalescent Fund acknowledges with grateful thanks further contributions towards "Nurse K.'s" travelling expenses to America, from the Lady Superintendent and Sisters Frances and Una, Private Hospital, Dublin, and Nurse E. B. Nurse K. starts on her journey next week, and wishes to thank very heartily and gratefully all those who have given her their help and sympathy. She would have liked to reply herself to each kind letter, but all her powers just now are occupied in learning how to walk again after many months in bed, in order to be ready for her voyage.

### The Victorian Era Exhibition.

THE Victorian Era Exhibition was duly opened on the 24th inst. Everything was in that delightful state of unpreparedness usual to exhibitions during the first few days of their existence. Everybody was eager to know something, or anything, if only the hour when H.R.H. the Duke of Cambridge would perform the ceremony; but the officials, in their livery of chocolate and gold, the policemen, not yet in their summer suits, were in a state of blissful ignorance, consequently people were hurrying hither and thither, vainly endeavouring to find out what was going to be done, and asking for the programmes which did not arrive until two o'clock. At a quarter-past twelve, however, the band struck up "God Save the Queen," the soldiers *en route* between the Earl's Court entrance and the Empire of India Theatre assumed a martial attitude in answer to the word of command, and His Royal Highness and suite came into sight. Then were the theatre doors flung open to the unfortunate many, unprovided with special tickets, and they crowded unceremoniously in, just in time to see the Duke conducted to the place of honour in the centre of the platform. The choir then sang Cowen's Commemoration Ode, accompanied by a band, after which a speech, inaudible except to the near bystanders, was read, and then His Royal Highness, after a few words expressing his pleasure at being present on this auspicious occasion, pronounced the exhibition open. Immediately the choir sang Sir Michael Costa's arrangement of the National Anthem, and the Duke left the platform in order to see (or not to see) the exhibits, the crowd following after.

The "Nursing Section" has been allotted the gallery immediately on the right upon entering the Empire of India Theatre from the Imperial Court, and promises to be, when complete, interesting, instructive, and, judging by the number of persons visiting it during the first hour after the opening, popular as well. It seems a pity, however, that only the Jubilee Nurses' Institute and the London Hospital should be represented; still one hospital chronicles the rise and progress of modern nursing, and when all is in order the public will have an opportunity of learning to what perfection the art of nursing has been carried, will have seen a few relics of bygone days, but will know nothing more of the intervening years during which the trained nurse of to-day has been evolved, as no attempt is visible to make the exhibition historical or widely representative of the many phases of nursing in the present day.

The contrast between the trained nurse and her prototype is aptly illustrated by a couple of wax models, dressed by one of the "Sisters" of the London Hospital, to portray "Mrs. Gamp" and her modern representative. The one in her untidy musty-looking dress and shawl, her rusty black bonnet, with her toilet and nursing impedimenta tucked into a brilliantly-coloured handkerchief, from which protrudes the neck of an unmistakable (in this case soda water) bottle, is some degrees more repulsive than the old lady that sits by the way-side and mends chairs; the other in her trim gown of delicate blue, her dainty hemstitched apron, her snowy half-sleeves and becoming cap, is a vision of freshness good to behold.

The building in which the nursing section stands is ark-shaped, with the windows in the apex of the roof. The slope between the glass and the walls is hung, tent fashion, with linen of broad alternating red and white stripes. The walls for about a third of their height from the roof are painted a delicate art-green, and the remainder is lined with the newly-invented glass tiles called "Opaline," of a harmonious, but more pronounced tone of colour. The flooring is spread with cork carpet of an inlaid wood pattern. The stands right and left at the entrance of the section are occupied by the "Queen's Nurses." That on the right is picturesquely fitted as a cottage, such as is commonly the scene of the labours of the nurses of this association. The furniture had not as yet arrived, nor had the artist finished painting the plenishings necessary even in the humblest home; but the little (wax) patient suffering from pneumonia was there as large as life, with hectic flush and drooping lids true to nature. A pleasant capable Jubilee nurse (not wax) was in attendance. Her uniform of dark grey-blue, her plain apron of strong linen, her simply-folded cap, looked so suitable for her work, as it was agreeable to the eye. It is easy to imagine how eagerly her patient would watch for her as her cycle ran swiftly down the country lane so invitingly portrayed in the background.

A beautiful wax nurse, got up in the latest style, and mounted on a cycle, was sent to be exhibited by Messrs. Debenham and Freebody; unfortunately her nurseship was quite unable to maintain her equilibrium, and, much to the amusement of her living prototypes, had to be carried off in an impromptu ambulance. She is to reappear as soon as she can be fixed up comfortably.

Models of many of the handy contrivances by which a district nurse replaces the orthodox appliances in out-of-the-way places may be found upon the table in the opposite stand; they are familiar to nurses, but must be a delightful surprise to many of the general public, who either do their nursing themselves, or who are fortunate to possess all the luxuries that money can buy. What could be simpler than the cradle necessary to protect a wounded limb than that made of the two halves of a hoop, fastened at the top with a screw, and yet it answers its purpose admirably.

Keeping to the left, we come to Messrs. Allen and Hanbury's stall, on which is a very large selection of instruments, &c. The opposite side was divided between the London Hospital and Messrs. Down's model of an operating theatre, fitted with all the recent improvements.

The London Hospital has sent no less than six life-size models of patients—four children and two adults. Each reposes in a white enamelled cot in an appropriate attitude. Two of the child models show the use of different splints in cases of hip-joint disease, and one is prepared for abdominal section. The old lady suffers from a broken leg, which is exquisitely bandaged and hung in an adaptable cradle: whilst the gentleman is supposed to be a case of typhus, with the temperature reduced by overhanging ice-trays, his woebegone and somewhat worn countenance exciting comments of a severely personal nature from the spectators. Crowded into the limited space are up-to-date tables and appliances of every description.

Messrs. Down's new operating table, in which the recently introduced glass top is replaced by one of zinc heated by hot-water pipes, is the most interesting of many seemingly perfect exhibits. It is more worthy of attention from the fact that within a few yards stands the operating table in use a hundred years ago at the London Hospital. In that the frame is of oak, the top leather, and the limited adjustments were evidently achieved by the operator putting his feet into one or other of the quaint leathern shoon at the end of the levers. One more relic claims the affectionate recognition of the nation, it is Florence Nightingale's travelling carriage of the Crimean War. Of wood and basketwork, waggon-like in shape, the back protected by a leathern hood, the front veiled by movable curtains, in that day of strife it was hailed as the harbinger of comfort by our wounded soldiers. The paint is faded to a dingy blue, the woodwork worm-eaten; we shudder as imagination brings before us pictures of its springless joltings over unbeaten tracks, yet we regard it with reverence as we note on every side the rich harvest sprung in most part from the seed sown by a simple English gentlewoman.

### Invalid Cookery.

### MENUS FOR DIABETIC PATIENTS.

ARTICLES of food containing starch or sugar must be steadily avoided by diabetic patients. At first sight it may appear a long list of "forbidden" food, but by no means need the sufferer feel condemned to what he fancies is unpalatable. A great variety can be obtained from the "allowed" list. Freshly-cooked meat is the most nourishing, but meat may be recooked in such a way as to retain the juices. At this time of year cold meat is not unwelcome; it is rendered especially palatable by laying the slices in a marinade of salad oil, a few drops of tarragon vinegar, a dust of coralline pepper and salt, and a little chopped parsley for about one hour; then arrange them neatly in a small dish, with a fresh-picked salad round. Cheese can be made into a variety of savouries, which may take the place of puddings. Some patients find a difficulty in digesting cheese, and in such a case a little bicarbonate of soda, a piece of butter, and a little milk should be cooked with it. It will be found useful to give a list of foods for diabetics, and we cannot do better than quote from Mrs. Ernest Hart's most explicit list.

### LIST OF ARTICLES OF FOOD.

*Allowed.*—Butcher's meat of all kinds; ham, bacon, and tongue, when not sugar cured; poultry and game; fish of all kinds; oysters and shellfish; crabs, lobsters; beef tea; broth not thickened; soups made of meat stock without starchy thickening; jellies made without sugar; aspic; tripe; German sausage; eggs, cheese, cream cheese, and cream; butter, fat, oil, and lard; caviare; almond cakes, bran cakes, and gluten bread, as substitutes for wheaten bread; "torrefied" or charred bread; saccharine to replace sugar; cabbage, endive, spinach, broccoli, Brussels sprouts, lettuce, spring onions, cucumber, green asparagus, water-cress, sorrel, salad, celery, tomatoes, artichokes, mushrooms, cauliflowers, seakale, turnips, French beans, vegetable marrow, dandelion, cardoons, mustard and cress, radishes, turnip tops, and nettles; unripe fruit, such as green goose-berries, green currants, and unripe apples cooked with saccharin; nuts of all kinds, except chestnuts; sardines in oil; pickles; savoury jelly; custard.

*Forbidden.*—Sugar in any form; wheaten bread, oatmeal cakes, porridge, ordinary biscuits, rice, arrowroot; potatoes, carrots, parsnips, beans, and peas; sago, tapioca, macaroni, vermicelli; Spanish onions; all sweet fruits such as grapes, cherries, peaches, strawberries, apricots, plums, gooseberries, currants, oranges, and all preserved fruits; pastry; puddings of every kind which contain sugar or farinaceous foods; beetroot; liver; English sausages; treacle.

—*Diet in Sickness and in Health.*

### MENUS.

SUNDAY.

*Breakfast.*—Poached eggs on very dry toast, cold ham.
*Dinner.*—Roast fowl garnished with watercress, grilled mushrooms, green gooseberry fool sweetened with saccharin.
*Tea.*—Fillets of fresh haddock with tomato purée, gluten bread and butter.

MONDAY.

*Breakfast.*—Grilled fowl, boiled egg.
*Dinner.*—Fillets of beef, green asparagus, cream cheese and watercress.
*Tea.*—Scolloped chicken, bran scones.

TUESDAY.

*Breakfast.*—Fried fillets of sole, cold bacon.
*Dinner.*—Fish soup, roast lamb, mint in aspic jelly, spinach, devilled diabetic biscuits.
*Tea.*—Stuffed tomatos, potted shrimps, gluten bread and butter.

WEDNESDAY.

*Breakfast.*—Buttered eggs on very dried toast, hot rolled ham.
*Dinner.*—Lobster salad, boiled fowl with cream sauce thickened with almond flour, cheese soufflé.
*Tea.*—Hot tripe stewed in milk, cold lamb marinaded.

THURSDAY.

*Breakfast.*—Fried smelts, cold tongue.
*Dinner.*—Boiled calf's head, spring cabbage, custard pudding.
*Tea.*—Savoury eggs and cress, cold ham.

FRIDAY.

*Breakfast.*—Tongue toast, omelet.
*Dinner.*—Mutton cutlets, hot tomatos, almond flour pudding.
*Tea.*—Fish soufflé, cold spiced beef.

SATURDAY.

*Breakfast.*—Eggs sur le plat, grilled kidneys.
*Dinner.*—Clear soup, curry without rice, cheese pudding.
*Tea.*—Dressed crab, almond flour cake.

RECIPE FOR EGGS SUR LE PLAT.—Butter a small pie dish or fireproof one, then carefully break the eggs into it, season with a little salt and coralline pepper, add one dessert-spoonful of fresh cream to each egg, and put a small piece of butter on each; place the dish on a tin containing hot water and stand in the oven for eight minutes, when the whites should be set; sprinkle with a little chopped parsley before serving.

CHEESE PUDDING.—Break two eggs into a basin and mix them with a little salt, pepper, and one ounce of grated Parmesan cheese and a quarter of a pint of milk; pour the custard into a buttered pie dish and put a few pieces of butter on the top; place the dish in a tin containing boiling water, and cook the pudding in a very moderate oven for twenty minutes or till the pudding is set.

Recipes for any of the above dishes can be had by enclosing seven stamps to "Dietetics," THE HOSPITAL Office, 28 & 29, Southampton Street, Strand, W.C.

### Novelties for Nurses.

### THE "HOLDFAST" SYRINGE.

IT is by care for small things that success is generally attained. Few things can be more aggravating, and in some cases more dangerous, in the administration of an enema or a douche than to find that the tail of the instrument has slipped out of the water and that one is injecting air instead of fluid. Yet this is an accident which is liable to happen to anyone in using the ordinary enema syringe. It is not always easy to keep an eye on both ends of the tube, and if the end which lies in the basin happens to slip out of the water air is sure to be injected. Nurses, then, have cause to be grateful to

Messrs. Bailey for their admirable "holdfast" syringe, an enema with a rubber suction end which, by simple pressure, will stick like a boy's sucker to the basin, and so keep the end always under water. It is only necessary to press the lozenge-shaped end against the basin to cause it to adhere tenaciously. Thus the nurse's attention need no longer be directed to the position of the syringe, which can be used freely and conveniently, and the whole of the solution utilized without trouble. The absence of all metal renders the syringe a safe medium for perchloride of mercury injections. The prices are very moderate. The syringes can be obtained at 38, Oxford Street.

### Everybody's Opinion.

[Correspondence on all subjects is invited, but we cannot in any way be responsible for the opinions expressed by our correspondents. No communication can be entertained if the name and address of the correspondent is not given, or unless one side of the paper only is written on.]

### BADGES FOR NURSES.

MRS. MUMFORD, secretary, Nurses' Co-operative Association, Ipswich, writes: In reference to "A. L. M.'s" letter respecting the abuse of uniform, the Ipswich Nurses' Co-operative Society has adopted a badge in the shape of a bronze medal, with the name of the association on it, attached by a cardinal ribbon to the indoor and outdoor uniform. This at once guarantees they are thoroughly trained nurses.

### NARROW-MINDED NURSES.

"AN INTERESTED READER" writes: Having seen an article in THE HOSPITAL some little time ago on "The Narrow-mindedness of Nurses" I should like to venture an opinion on the subject. I must admit it is a common failure amongst nurses to talk "shop" as it is called. In the majority of cases, however, they cannot help it. For instance, when speaking to residents—and nurses as a rule have not much time for talking "shop" abroad—if a nurse were to converse on any topic of the day she would be looked on as quite a phenomenon, and if attended to would probably be answered in monosyllables, surely not because the subject is new to the auditors. I am afraid that doctors are too fond of discussing nurses to one another, and could be told with advantage to "go and read the newspaper," as the nurses were told to do in your pages.

### ALMSHOUSES FOR NURSES.

"AN OLD NURSE" writes: "I think if the public knew how hard is the case of old nurses like myself they would give us their help and sympathy. Many of us between sixty and seventy years of age are still most anxious to work for our living, yet time goes on and our cases get fewer and fewer and we cannot get fresh ones, not because we feel too old for work, but because we are considered to be so. It is natural, I know, that younger faces and voices should be more attractive to patients, yet it is very hard, as after years of work our chances of earning our living become less and less, and we have only before us the miserable anticipation of having nowhere to lay our heads when our means of income stops altogether. If nurses' almshouses could be instituted, however humble, it would be a great comfort; but it is not for me to suggest what should be done. I simply wish to state, not only my own case, but the case of all those who have worked hard in their profession and are getting old, and to whom the prospect of the future is a matter for continual dread."

### NURSES AND BICYCLES.

A CORRESPONDENT writes: Bicycles may or may not (decidedly not I should say) be suitable to women, but surely if nurses are following the rest of their sisters in the "bike" craze would it not be better to leave their uniforms at home, and so not bring discredit and disrespect on their profession. Last Saturday I saw two nurses in Guy's uniform with a "bike" on London Bridge platform. As a man I felt extremely sorry to think what our nurses were coming to—they who should be a type of all that is best in women descending to the lower level of the advanced women of to-day.

"A WOMANLY WOMAN" writes? As a nurse of many years' standing, may I make my protest through your columns against women of my profession making themselves so ridiculous and conspicuous by going out with their bicycles in their nursing uniform. Last Saturday afternoon I saw on the platform of London Bridge Station two nurses (who I presume from their bonnets were Guy's) with a bicycle. Anything more objectionable than an unwomanly woman cannot be imagined. In my young days in the profession we were proud of working quietly and unknown. Now nurses must be first and foremost in every amusement, magazine or newspaper. Why should these things be more necessary to the happiness and health of a nurse now when we got on so well without them? If a woman cannot give herself heart and soul to the work she has taken up, as we have done, let her stay at home, where she can enter into these amusements without dragging an honourable profession through the mud.

### REGISTRATION OF MIDWIVES.

MRS. WALLACE BRUCE, Hon. Secretary of the Association for Promoting Compulsory Registration of Midwives, writes: Our attention has been called to an inquest, held in Hertfordshire by Dr. Lovell Drage, on a woman who had died in childbirth after having been attended by a midwife holding the diploma of the London Obstetrical Society. This case, already, no doubt, known to all your readers, is one of the strongest possible arguments in favour of our contention that midwives should be registered as well as trained. It is one of the rules of the London Obstetrical Society that in a serious case like the one in question medical help should be called; but in the absence of any Registration Act no penalty can be inflicted for neglect of this rule. The midwife is, in fact, responsible to no one, and under these conditions the fact of her holding a diploma or certificate may be a source of danger rather than of protection to the public. We contend that is a most dangerous position, and urge strongly that training and registration must go hand in hand, and we are confident that when the community is a little more alive to the dangers of the present system the legislation necessary to secure these will soon be an accomplished fact.

### Workhouse Infirmary Nursing Association.

THE annual meeting of the Mary Adelaide Nurses is always enjoyable, and Tuesday's "gathering" at the Portman Rooms, Baker Street, was no exception to the rule. There were perhaps fewer nurses than usual, for many in country unions find it difficult to spare time to come up to London for a night, but those who were there spent a thoroughly pleasant evening. Miss Twining, of course, was present, Miss Wilson, Miss Gill, Miss C. J. Wood, Miss Paget, Miss Fynes Clinton, Miss Brierly, Miss Gibson, Miss de Pledge, Miss Moir, Dr. Cullingworth, Dr. and Mrs. Humphreys, and Dr. Savill. Miss Gibson, matron of the Birmingham Infirmary, gave a short address, which was listened to with much interest, and afterwards Madame Rossiter gave a number of songs and recitations. It is pleasant to be able once again to congratulate the association on the appearance of its nurses, who looked as neat and nurse-like as could be wished.

### Where to Go.

IMPERIAL INSTITUTE, S.W.—On Thursday, June 3rd, at 3.30 p.m., Mr. Bancroft will give his well-known reading of Charles Dickens's "Christmas Carol" at the Imperial Institute, in aid of the Colonial Nursing Institute. Tickets, half a guinea each, may be obtained from the hon. secretary, Mrs. Francis Piggott, at the Imperial Institute, or from Mrs. Joseph Chamberlain, 40, Prince's Gardens, S.W., Mrs. E. Wingfield, 40, Albion Street, W., Lady Musgrove, Hurst-on-Clays, East Grinstead, or the Dowager Lady Westbury, 134, Cromwell Road, S.W. Princess Henry of Battenberg has given her patronage.

### For Reading to the Sick.

ASCENSION TIDE.

"AND when He had spoken these things, while they beheld, He was taken up; and a cloud received Him out of their sight."—*Acts* i. 9.

**Verses.**

Hail the day that sees Him rise
On His throne above the skies;
Christ the Lamb, for sinners given,
Enters now the Highest Heaven. Alleluia!

Lo! the heaven its Lord receives,
Yet He loves the earth He leaves;
Though returning to His throne,
Still He calls mankind His own. Alleluia!

—*C. Wesley.*

Christ has raised our human nature
On the clouds to God's Right Hand;
There we sit in heavenly places,
There with Him in glory stand.
Jesus reigns, adored by angels;
Man with God is on the throne;
Mighty Lord, in Thine Ascension
We by faith behold our own.

—*Bishop C. Wordsworth.*

Our great High Priest and Shepherd Thou
Within the veil art entered now,
To offer there Thy precious blood
Once poured on earth a cleansing flood.

And thence the Church, Thy chosen Bride,
With countless gifts of grace supplied,
Through all her members draws from Thee
Her hidden life of Sanctity. —*Latin Hymn.*

**Reading.**

Truly, if we could ever live in this day, all were joy. It It is the crown of all joys, the joy of all creation, the warder of the blessed angels, the union of all being, the family of the earthly course of the Son of God, His entrance into glory. He ascended, not into the highest heavens only, but far above all heavens. There, where no creature is or can be; there, encircled, embosomed, impenetrable with the Godhead, adored together with the Godhead by all creation, is the body of Christ, our God, our King, our Head; who calls us "His body," calleth us brethren!—*E. B. Pusey.*

I ascend unto my Father, and your Father; and to my God, and your God.—*St. John* xx. 17.

Yet although in body for us at God's right hand, He is still, as God, as near to us as when He was in the flesh. Here where we are gathered in His Name, could our eyes behold Him, He is in the midst of us. He is with us unto the end of the world. Yea, He is with us in a nearer way, if we will, dwelling in us by His Spirit, and feeding us with His Body and His Blood.—*E. B. Pusey.*

Be ye lift up, ye entrances of eternal life, of renunciation of the world, and conversion to God. And the King, in whom we may glory without pride, shall come in, who hath overcome the gates of death, and having opened for Himself the heavenly places, fulfilled that which He said, "Be of good cheer, I have overcome the world."—*St. Augustine.*

### More Nurses for Greece.

FOUR more nurses have been sent out to Athens this week by the *Daily Chronicle* Fund. They are Miss Jane C. Child, trained at St. Thomas's Hospital and late matron of the Lewes Hospital; Miss Emma Dobson, trained at the Norfolk County Hospital; Miss Annie Victoria Latham, trained at St. Bartholomew's Hospital; and Miss Sarah E. Collins, trained under St. John's House at the Metropolitan and other hospitals. These nurses left Charing Cross on Wednesday morning. Twenty-seven English nurses have now been sent out by the fund.

### Notes and Queries.

*Nurses for Bermuda.*

(266) Having seen that two nurses will be wanted to go to Bermuda, I should be glad to know something about the posts in question for myself and a friend. We are fully trained nurses.—*S. M. A.*

See reply to similar question in this column on May 15th.

*Fever Training.*

(267) I am a trained medical and surgical nurse, and wish to give special attention to fevers for a few months. Can you tell me where to apply? I am afraid the M.A.B. hospitals do not take nurses for a short term.—*Sara.*

You had better apply at some of the M.A.B. hospitals, or answer the advertisements for nurses which are constantly appearing in our columns. We do not think that the M.A.B. require nurses to bind themselves for any lengthened period of service. Few nurses care to take up fever work for a permanency, and the trained nurses who obtain appointments at the various fever hospitals are usually those who, like yourself, wish to gain experience in that special branch of nursing. Study the advertisements in last week's HOSPITAL.

*Private Nursing.*

(268) Can you tell me of any institution which I could join for private work, living at home? I have had nine years' hospital work, and have been now private nursing for two years.—*Nurse C.*

You omit to mention where "home" may be, in London or the country. We should think you had better advertise. In joining any private nursing institution be very careful to ascertain that it is a *bona fide* one, and not, as many of these so-called "co-operations" in reality are, a mere private speculation.

*Patenting.*

(269) Can you tell me how to patent an article? Must I have a model first made?—*M. F.*

Apply at the Patent Office, 25, Southampton Buildings, W.C.

*Hospitals in Italy.*

(270) Are there any English hospitals in Italy, and are they worked on the same principles as English ones?—*Nurse T.*

There is an English matron at the International Hospital at Naples. We do not know of any exclusively English hospital in Italy.

*Homes for the Blind.*

(271) A young widow, daughter of a clergyman, dependent upon her own exertions, has a boy of seven, blind from birth, and lately paralysed in lower limbs. Can you help me to a home where he could be educated so far as his infirmities will allow?—*N.*

You had better consult the lists of institutions for the blind which you will find in Burdett's "Hospitals and Charities" (Scientific Press, 28 and 29, Southampton Street, Strand), and write direct to any of these that seem to meet the circumstances of the case. You do not say if the home should be in London or the country.

*Training.*

(272) I want to enter a general hospital as probationer, and would be glad to know if being a housemaid would be considered an objection.—*A. M. L.*

In some hospitals it would be a recommendation. For instance, at the Middlesex Hospital (London, W.) Miss Thorold prefers her non-paying probationers to have been in domestic service.

*Bicycling.*

(273) Can you tell me if it is proper for a nurse to ride a bicycle in her uniform, and if there would be any objection to a neat dark blue uniform without veil? I am a private nurse, and off-duty time is so limited I grudge minutes spent in changing clothes. How do the members of the Guy's Cycling Club manage? I shall be grateful for advice.—*One Who Respects Her Uniform.*

We do not like to see a nurse riding in uniform, unless she is a district nurse and using her bicycle as a matter of business. In every way it is far better to change, for you do not want to make the dress you are wearing in the sick room dusty and soiled, nor is it advisable to ride in a skirt long enough and full enough to look nice in the house. A coat and skirt (of some plain, serviceable stuff) are quickly got into. The Guy's nurses all wear the club uniform, which consists of a navy blue coat and skirt, with cotton or woollen shirt, and black or white sailor hat with black ribbon or Guy's colours.

*Training.*

(274) Please tell me whether it is advisable to enter a general hospital or a workhouse infirmary for training. I am twenty-one; and have just finished two years fever training, but fear I may not find it easy to enter a recognised training school so young.—*Mary G., Liverpool.*

Unless you wish to come to London, why not apply at the Mill Road Infirmary, Liverpool, where excellent training is given? Whether you enter a general hospital or a workhouse infirmary would depend upon what are your ambitions for the future. Few general hospitals will take probationers under twenty-four or twenty-five, and if you can get into a good infirmary training school at once you would be wise to do so.

*Home Wanted.*

(275) I am anxious to find a home for an old lady aged 79, not an invalid. Not more than £1 a week can be paid for her. Can you help me with any advice? Is there any cheap edition of "Hospitals and Charities"?—*Sister W.*

Have you answered any of the advertisements of "Residential Homes" which appear each week in our columns? We should think you would have little difficulty in finding what you want, and it might be well to put in an advertisement yourself. You do not say if you prefer town or country. If the sea would suit, you might write to Miss Walker, Dudley House, Marine Parade, Worthing. If you will state a few more details we may be able to help you further. There is no cheaper edition of "Hospitals and Charities" than the 5s. one.

## Figures and Tables

**Figure f1:**